# Wheat Cultivars With Contrasting Root System Size Responded Differently to Terminal Drought

**DOI:** 10.3389/fpls.2020.01285

**Published:** 2020-08-19

**Authors:** Victoria Figueroa-Bustos, Jairo A. Palta, Yinglong Chen, Katia Stefanova, Kadambot H. M. Siddique

**Affiliations:** ^1^The UWA Institute of Agriculture, and UWA School of Agriculture and Environment, The University of Western Australia, Perth, WA, Australia; ^2^CSIRO Agriculture & Food, Wembley, WA, Australia

**Keywords:** terminal drought, root system size, water use efficiency, water use, wheat

## Abstract

In the Mediterranean-type environment of Australia and other parts of the world, end-of- season or terminal drought is the most significant abiotic stress affecting wheat grain yields. This study examined the response of two wheat cultivars with contrasting root system size to terminal drought and the effect of terminal drought on grain yield and yield components. The cultivars were grown in 1.0 m deep PVC columns filled with soil in a glasshouse under well-watered conditions until the onset of ear emergence (Z51) when well-watered and terminal drought treatments were imposed. Terminal drought reduced stomatal conductance, leaf photosynthesis, and transpiration rates faster in Bahatans-87 (larger root system size) than Tincurrin (smaller root system size). Terminal drought reduced grain yield in both cultivars, more so in Bahatans-87 (80%) with the large root system than Tincurrin (67%) with the small root system, which was mainly due to a reduction in grain number and grain size in Bahatans-87 and grain size in Tincurrin. In the terminal drought treatment, Bahatans-87 had 59% lower water use efficiency than Tincurrin, as Bahatans-87 used 39% more water and reduced grain yield more than Tincurrin. The lesser reduction in grain yield in Tincurrin was associated with slower water extraction by the small root system and slower decline in stomatal conductance, leaf photosynthesis, and transpiration rates, but more importantly to faster phenological development, which enabled grain filling to be completed before the severe effects of water stress.

## Introduction

In the Australian grainbelt, annual rainfall declined from 1900 to 2009 by up to 20% and a further 10% reduction is estimated by 2070, threatening wheat production (Asseng and Pannell, 2013). This change in annual rainfall has followed a declining linear trend with annual wheat yield losses (up to 1.5%) in the Australian grainbelt from 1981 to 2018 (Ababaei and Chenu, 2019). End-of-season drought or terminal drought occurs in most Australian wheat-growing areas (Chenu et al., 2013), particularly in Mediterranean-type environments, where crops are usually sown on the first major rainfall between mid-April and the end of June. In this region, 80% of annual rainfall is received between May and October and soil water losses by deep drainage below the root zone can occur during early winter in deep sands when the crop is small (Scanlon and Doncon, 2020). Conversely, water deficits develop after flowering when rainfall decreases and evaporation increases (Turner and Nicolas, 1987). Terminal drought reduces grain yield mainly because it reduces photosynthesis and the duration of grain filling (Fabian et al., 2011; Saeidi and Abdoli, 2015; Christopher et al., 2016; Yu et al., 2016). Under terminal drought, every millimeter of extra soil water extracted by the root system is critical for maintaining grain filling and improving water use efficiency (Passioura, 1983).

The root system plays a key role in the uptake of soil water and regulation of leaf senescence and leaf photosynthesis rate during grain filling (Kong et al., 2013). A deeper root system can prevent a severe water deficit from developing by accessing soil water at depth during terminal drought (Palta and Watt, 2009). Root length and root biomass are positively correlated with water use (Abdolshahi et al., 2015). However, a compact, deeper, and uniform root system should reduce water use early in the season increasing water availability from deeper soil layers post-anthesis (Palta and Turner, 2018). This is because conservative water use during vegetative growth will increase available soil water at flowering and during grain filling, and thus increase harvest index and grain yield (Araus et al., 2002; Ahmed et al., 2018). In contrast, large and shallow root systems can extract water from the top layers of the soil profile during vegetative growth, when rainfall is plentiful in the winter season (Manschadi et al., 2006). Cultivars with larger root systems had greater grain yield than cultivars with smaller root systems in rain-fed experiments in Central Europe (Motzo et al., 1993; Středa et al., 2012). Early season drought reduced grain yield less in Bahatans-87 (larger root system size) than Tincurrin (smaller root system size) due to slow phenology that allows better recovery in leaf area and shoot biomass at anthesis (Figueroa-Bustos et al., 2019). However, it is not clear whether a strategy of higher water use (higher biomass production and transpiration rate) of the cultivar with larger root system size or the saving water strategy (lower water use combined with a lower leaf area) of cultivar with smaller root system size are superior under terminal drought. This study examined whether differences in root system size are associated with differences in tolerance to terminal drought. It was hypothesized that root system size (in terms of root length and biomass) at anthesis in a wheat cultivar with a large root system increase water use, water use efficiency, and grain yield relative to cultivars with smaller root system size, under terminal drought. To test this hypothesis, two wheat cultivars with different-sized root systems were exposed to terminal drought. Differences in shoot and root traits were analyzed before starting the water treatment and at final harvest.

## Materials and Methods

### Plant Material and Growing Conditions

**Figure 1:**
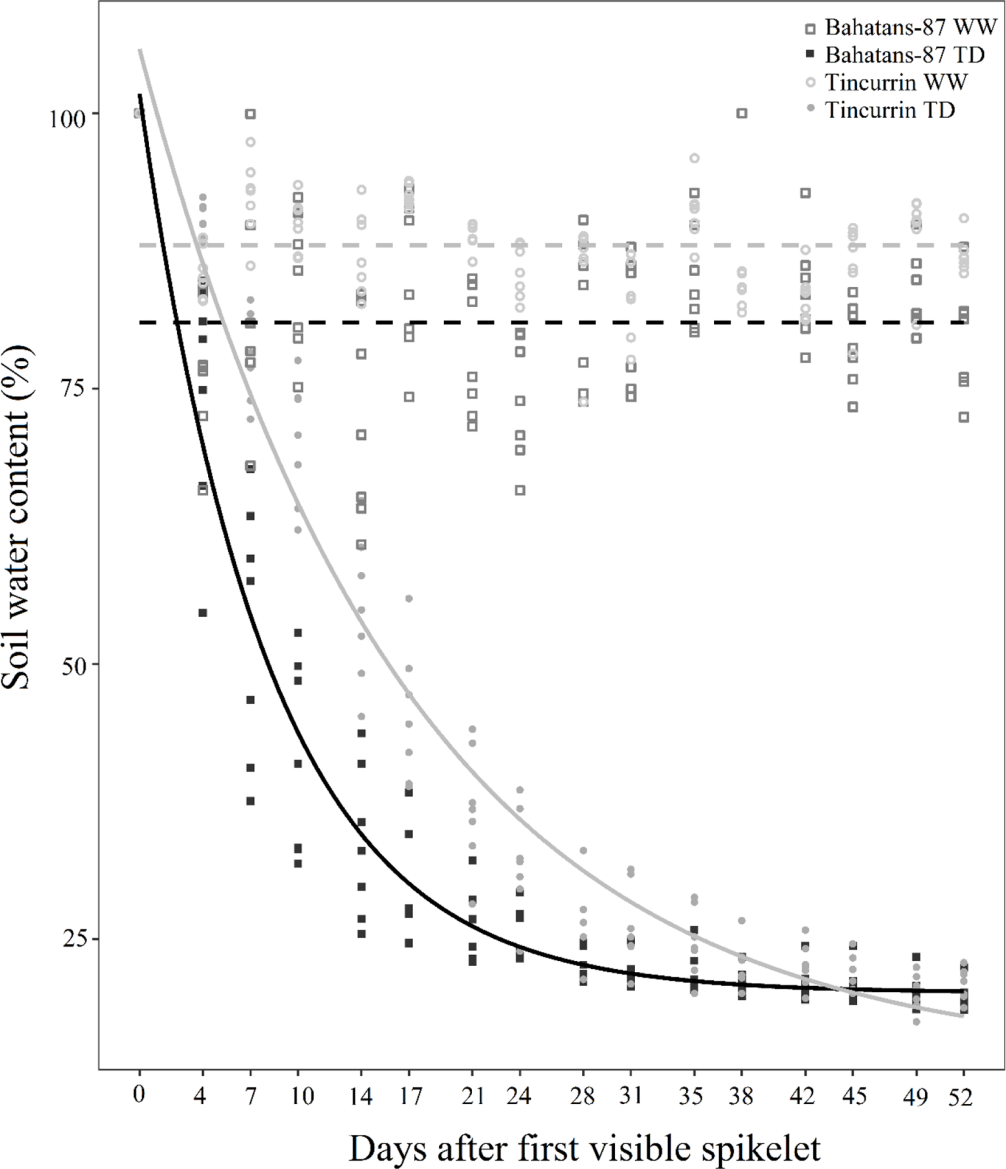
Change with time in soil water content (%) after the first spikelet on the main stem ear was visible (Z51) in Bahatans-87 (large root system) and Tincurrin (small root system) under well-watered (WW) and terminal drought conditions (TD). Points represent the data of each replicate individually. Broken lines represent soil water content in WW treatment. Solid lines represent the best exponential fitted model for soil water content over days after booting stage TD.

Two wheat (*Triticum aestivum* L.) cultivars—Bahatans-87 (large root system size) and Tincurrin (small root system size)—were selected from a phenotyping study characterizing root trait variability in 184 genotypes using a semi-hydroponic phenotyping platform (Chen et al., 2020), and further validated in a rhizobox experiment (Figueroa-Bustos et al., 2018). The root system size were categorized, following the definition of Hamblin and Tennant (1987); Palta and Watt (2009); Palta et al. (2011) and was based on total root length and root biomass. The selection of these two cultivars was based on previous studies (Figueroa-Bustos et al., 2018; Chen et al., 2020). Growth and development of the two cultivars with contrasting root system size were characterized in a previous study under well-watered conditions (Figueroa-Bustos et al., 2018) and the differences in root system size between the two cultivars were correlated with leaf area, tiller number, leaf biomass, and phenological development. The two cultivars are not near isogenic lines, and they do not have common parents or the same phenological development. Previous studies showed that tall and semi-dwarf isogenic lines with same phenology had similar root biomass, and root: shoot ratio and this is because there are a strong association between phenology and root system size (Siddique et al., 1990a). Bahatans-87 is an old bread wheat released in 1924 in Algeria and Tincurrin is a biscuit wheat released in 1978 in Australia. The two cultivars were grown in PVC columns (0.15 m diameter, 1.0 m deep) with a long sleeve clear plastic bag (150 µm thick with 24 small holes in the bottom) inserted into each column for the ease of root recovery at harvest. The pots (0.15 m diameter and 1.0 m deep) contained 26 kg of soil (volume 0.01766 m3). Several studies have reported that the above volume of soil and particularly depth (1 m) did not show root development restrictions in wheat (Aziz et al., 2016; Saradadevi et al., 2017; Figueroa-Bustos et al., 2019; Pooniya et al., 2019). In addition, Turner (2018) suggested that large tall pots better simulate water extraction and root development similar to field conditions than smaller pots. In our experiment, the size of the pot simulated a similar rate of decline of soil water content (**Figure 1**) that observed under field conditions in Western Australia. Small holes at the bottom of the plastic bag to facilitate drainage from the plastic bag to the PVC pot, which had fixed bottom lid and short plastic tubes connected to a bottle to collect any drainage. Drainage was minimized by manually watering each pot. Each column was filled with soil at a bulk density of 1.47 g cm^−3^ over a 5-cm layer of gravel at the bottom to facilitate drainage. The soil was a reddish-brown sandy clay loam (Red Calcic Dermosal) (Isbell, 1993), obtained from the top 0 to 15 cm of a field site at Cunderdin (31°64’ S, 117°24’ E), Western Australia. The soil consisted of 63.5% brown sand, 8.3% silt, and 28.3% clay with a pH 6.0 (measured in a 1:5 suspension of soil in 0.01 M CaCl_2_). Air-dried soil was sieved to 2 mm and mixed with coarse river sand (200–2000 µm particle size) in a 4:1 ratio by weight using a cement mixer for uniformity (Aziz et al., 2016; Figueroa-Bustos et al., 2018). Fertilizer equivalent to 60 kg ha^−1^ N, 77 kg ha^−1^ P, 71 kg ha^−1^ K, and trace amounts of micronutrients (S, Cu, Zn, Mo, and Mn) was mixed homogeneously into the top 0.1 m soil layer in each column the day before sowing. The fertilizer dose at sowing corresponded to the optimal nutrient supply for wheat crops in the Cunderdin district in Western Australia, from where the soil was collected for the experiment (Flower et al., 2012). Five seeds per column were sown on 14 May 2020 and thinned to two plants per pot at the 1 to 2 leaf stage. At tillering (Z23) (Zadoks et al. (1974), 2.5 cm layer of plastic beads was uniformly spread on the top of the soil in each pot to prevent the soil water losses from evaporation. A water soluble fertilizer (Scott Peter excel) with 15% nitrogen, 2.2% phosphorus, 12.4% potassium, and other micronutrients was supplied through irrigation at 34, 49, and 65 DAS to prevent any nutrient deficiencies.

Plants were grown in an evaporative cooled glasshouse at The University of Western Australia, Perth, Australia (31°93’ S, 115°83’ E) from May to November 2019, with an average air temperature of 17°C (range, 9–27°C), relative humidity of 60% and natural daylight (photoperiod) of 11 to 12 h. From sowing to ear emergence, before the terminal drought was induced, all plants in each cultivar were watered twice a week to maintain the pot soil water capacity close to 80%. On each occasion, pots were weighed, and the amount of water supplied was based on amount of water transpired.

### Experimental Design and Treatments

A randomized complete block design was used with two factors and seven replicates per treatment, for a total of 28 columns (experimental units). Twelve extra columns (six columns per cultivar) were grown for measurements of root and shoot traits at the time when the first spikelet on the main stem ear was visible in each cultivar (Z51), just before the watering treatments were imposed. Five of the six replicates were used as controls. The layout of the experimental units in the glasshouse consisted of four columns by ten rows. One replicate consisted of two columns by two rows. The two watering treatments were imposed when the tip of the ear was just visible in the main stem of 50% of the plants (Z51; start of ear emergence). Water deficit was induced by withholding water from 14 columns (seven per cultivar) in the terminal drought treatment. A second group of seven columns per cultivar were well watered twice weekly to maintain above 80% of column water holding capacity (WW) until physiological maturity (Z91). The column water holding capacity was 4.6 L.

### Measurements

Phenological development was monitored regularly using the scale of Zadoks et al. (1974). Days after sowing (DAS) to boot swollen (Z51), anthesis (Z61), and physiological maturity (Z91) were recorded. Apparent duration of grain filling was calculated as the difference between days to physiological maturity and anthesis.

When the first spikelet on the main stem ear was visible in each genotype (Z51), just before inducing terminal drought, five columns from each cultivar were randomly selected and harvested. Plants were harvested by cutting the shoots from the roots at the crown, the number of tillers recorded, and stems and leaves separated for measurements of leaf area, leaf biomass, and specific leaf area (leaf area per unit leaf weight, SLA). Leaf area was measured using a portable leaf area meter (LI-3000, Li-COR Bioscience, Lincoln, NE, USA). Stems and leaves were dried separately in an oven at 60°C for 48 h and then weighed for shoot biomass.

Immediately after harvesting the shoots, the plastic bag inside each column was pulled out from the column and opened to recover the roots. The soil profile was sampled in 0.2 m sections from the top by cutting the soil with a carbon steel blade. The roots in each 0.2 m section were recovered from the soil by washing through a 1.4 mm sieve to produce a clean sample (Palta and Fillery, 1993). The recovered roots from each 0.2 m soil section were placed in plastic bags at 4°C until being scanned at 400 dpi per mm (Epson Perfection V800, Long Beach, CA, USA) to measure root length. The root samples were dried after scanning as per the shoot samples to measure root biomass and specific root length (root length per unit of biomass; SRL). Root images were analyzed using WinRHIZO Pro Software (v2009, Regent Instrument, Quebec, QC, Canada) (Chen et al., 2016). Specific root length, an indirect measure of the thickness of the root system, was estimated as total root length divided by total root biomass (Liao et al., 2006; Aziz et al., 2016).

From the time the first spikelet on the main stem ear was visible in each genotype (Z51) until the flag leaf in plants under terminal drought conditions had dried and rolled, measurements of stomatal conductance (g_s_), leaf net photosynthesis rate (P_n_), and transpiration (Tr) were made at 3- to 4-day intervals. Measurements were made on the top fully expanded leaf of the main stem on seven replicate plants between 10:00 am and 1:00 pm on days with clear sky using a LI-COR gas-exchanged system (LI-6400, LI-COR Bioscience, Lincoln, NE, USA) with LED light source on the leaf chamber. In the LI-COR cuvette, CO_2_ concentration was set to 380 µmol mol^−1^ and LED light intensity 900 µmol m^−2^ s^−1^, which is the average saturation intensity for photosynthesis in wheat (Austin, 1990).

The amount of water applied to each column at watering was recorded. Pre-ear emergence water use was calculated as the sum of water applied until ear emergence (Z51). After the well-watered and terminal drought treatments had commenced, the columns in both treatments were weighed twice a week. After weighing the columns, the soil in the well-watered treatment was slowly watered to keep the soil close to full capacity, while the columns in the terminal drought treatment were not watered. Post-ear-emergence water use was calculated as the difference in weight of individual columns at ear emergence (Z51) and at maturity plus the water applied in-between. Total water use was calculated as the sum of pre- and post-ear-emergence water use. The ratio pre-to post-anthesis water use was calculated. Water use efficiency (WUE_grain_) was calculated as grain yield per unit total water used. Soil water content (SWC) was calculated as

$$[1-({\text{W}}_{\text{c}}-{\text{W}}_{\text{n}})/({\text{W}}_{\text{c}}-{\text{W}}_{\text{d}}))]*100$$

where W_c_ is the initial column weight at saturation, W_n_ is the weight of the column on the day of measurements, and W_d_ is the weight of the column with dry soil.

Final pot weight of the droughted plants, when the plants were permanently wilted (w_f_), was recorded before harvest to calculate fraction of transpirable soil water (FTSW) (w_n_ − w_f_)/(w_i_ − w_f_), where w_n_ is the weight of the pot on the day of measurement, and w_i_ is the initial pot weight at saturation (Sinclair and Ludlow, 1986).

At final harvest, the number of tillers and spikes per plant was counted. Spikes were separated from shoots, oven dried at 60°C for 48 h before being hand-threshed. The number and weight of grains per plant were recorded. Harvest index (HI) was calculated as the ratio of grain yield to shoot biomass.

### Statistical Analysis

One-way ANOVA was used for the analysis of growth parameters collected at the beginning of ear emergence before the water treatments were imposed. The analysis aimed to compare the two cultivars at growth stage.

Two-way ANOVA was undertaken for the following response variables: days to anthesis, physiological maturity, growth and yield parameters at final harvest. The main effects of “cultivar,” “water treatment,” and their interaction were fitted in the model. The predicted means for the significant terms in the model were compared using least significant difference (LSD) at 5%.

Both ANOVA models accounted for the blocking structure, presented here as a replicate.

Measurements of stomatal conductance (g_s_), leaf net photosynthesis rate (P_n_) and transpiration (T_r_) were taken at days 7, 10, 14, 21 and 24 after the drought treatment started. The data were analyzed in two ways addressing two questions of interest. First, repeated measures techniques were used to assess the significance of the main effects of cultivar, water treatment, and their interaction along with fitting an unstructured model to account for the correlation of the observations measured on the same experimental unit for each of the 7 days. The second model modeled the dynamics of g_s_, P_n_, and T_r_ in time for the four cultivar and water treatment combinations. An exponential curve of type *y=a+br^x^* was fitted for each of the response variables, where *a*, *b*, and *r* are the shift, scale, and rate parameters, respectively, and *x* is the numbers of days. The same type of exponential curve was used to model the soil water content (SWC).

The data were analyzed using the statistical software Genstat statistical 20th edition (VSI International, Hemel Hemstead, UK, 2019).

## Results

### Phenology

The first visible spikelet on the main stem ear (Z51) appeared 29 days earlier in Tincurrin than Bahatans-87 (*P*< 0.01; **Table 1**). Regardless of water treatment, anthesis in Tincurrin occurred 13 days earlier than Bahatans-87. Terminal drought accelerated physiological maturity by 17 days (*P*< 0.01; **Table 1**) in both cultivars. Regardless of water treatment, grain filling took around 14 days longer in Tincurrin than Bahatans-87 (*P*< 0.01; **Table 1**). Terminal drought conditions shortened grain filling by 15 days in Bahatans-87 and 17 days in Tincurrin (*P*< 0.01; **Table 1**). The non-significant interaction between cultivar and treatment indicates that cultivars responded similarly to terminal drought.

**Table 1:** Number of days to the first visible spikelet on the main stem ear (Z51), anthesis (Z61), physiological maturity (Z91), and duration of the grain filling in Bahatans-87 (large root system) and Tincurrin (small root system) under well-watered (WW) and terminal drought conditions (TD) from the time the first spikelet on the main stem ear was visible.

Cultivar	Treatment	First visible main steam spikelet(DAS)	Anthesis(DAS)	Physiological maturity(DAS)	Grain filling duration(Days)
Bahatans-87	TD	101a	109a	131c	22c
	WW	100a	109a	147a	37b
Tincurrin	TD	72b	86b	121d	35b
	WW	72b	86b	138b	52a
*P* value (LSD)					
Cultivar		***(2)	***(2)	***(2)	***(2)
Treatment		NS	NS	***(2)	***(2)
Cultivar × treatment		NS	NS	NS	NS

Means followed by different letters differ significantly (LSD test). *** significant at P < 0.001. NS, not significant P > 0.05.

### Soil Drying, Stomatal Conductance, Leaf Rate Photosynthesis and Transpiration Rate

In the first 52 days of treatments, soil water content in the well-watered treatment was maintained at about 81% in Bahatans-87 and 88% in Tincurrin (**Figure 1**). The fitted curve of the soil water content model under terminal drought explained 96% of the variance (*P*<0.001). The fitted curve is presented as SWC = 20.15 + 81.62 × 0.88^day^ for Bahatans-87 and SWC = 13.86 + 91.97 × 0.94^day^ for Tincurrin (**Figure 1**). The dynamic of the curve showed that the soil water content under Bahatans-87 decreased faster than Tincurrin when water was withheld completely from ear emergence (Z51) (*P*< 0.001). The soil water content rate decreased 7% faster in Bahatans-87 than in Tincurrin (**Figure 1**) from the commencement of water stress treatment (ear emergence Z51) to physiological maturity. Three-gas exchange parameters measured in the study declined with increase in water stress. There was no difference between the cultivars in their gas exchange response to water stress (**Figure S1**).

**Figure 2:**
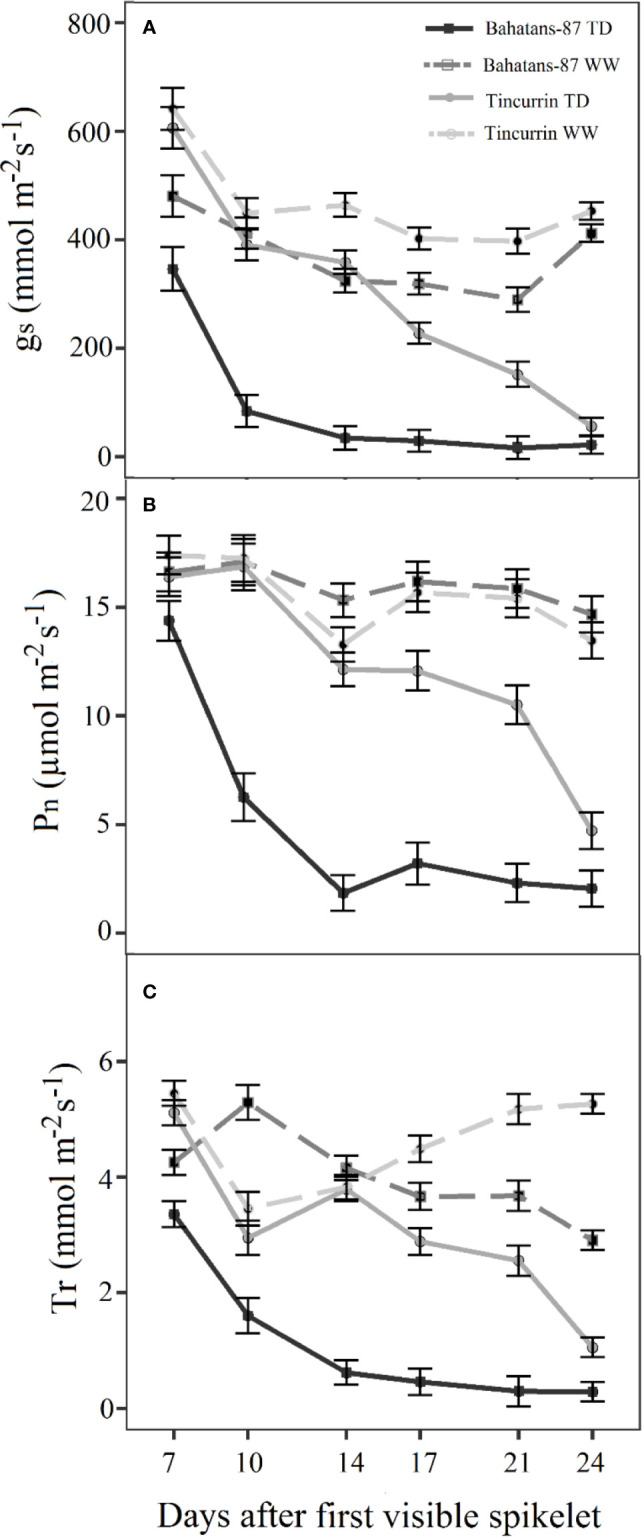
Change with time in **(A)** stomatal conductance (g_s_), **(B)** leaf photosynthesis rate (P_n_) and **(C)** transpiration rate (T_r_) after the first spikelet on the main stem ear was visible (Z51) in Bahatans-87 (large root system) and Tincurrin (small root system) under well-watered (WW) and terminal drought conditions (TD). Error bars represent standard errors of means (s.e.m.) (n=7).

Under well-watered conditions, stomatal conductance (g_s_) ranged from 290 to 481 mmol m^−2^ s^−1^ in Bahatans-87 and 398 to 641 mmol m^−2^ s^−1^ in Tincurrin (**Figure 2A**). Under terminal drought, g_s_ decreased from 346 to 17 mmol m^−2^ s^−1^ in Bahatans-87 and 607 to 56.3 mmol m^−2^ s^−1^ in Tincurrin in the first 24 days after the start of the treatment. The reduction in g_s_ under terminal drought was faster in Bahatans-87 than Tincurrin, with g_s_ decreasing by 90% in Bahatans-87 and 24 days in Tincurrin after 14 days of withholding watering (*P*<0.001). The best-fitted model explained 86% of the variance (*P*<0.001). The curves generated by the model for well-watered conditions followed the equations: g_s_ = 334.6 + 1682 × 0.717^day^ for Bahatans-87 and g_s_ = 427.3 + 20391 × 0.527^day^ and for Tincurrin. The curves for terminal drought conditions followed the equations: g_s_ = 18.8 + 7014 × 0.6551^day^ for Bahatans-87 and g_s_=−255 + 1297 × 0.9438^day^ for Tincurrin, indicating that g_s_ in Bahatans-87 declined 31% faster than Tincurrin under terminal drought.

The leaf photosynthesis rate (P_n_) under well-watered conditions ranged from 15 to 17 µmol m^−2^ s^−1^ in Bahatans-87 and 13 to 17 µmol m^−2^ s^−1^ in Tincurrin (**Figure 2B**), while under terminal drought it decreased from 14 to 2 µmol m^−2^ s^−1^ in Bahatans-87 and 16 to 5 µmol m^−2^ s^−1^ in Tincurrin (**Figure 2B**). The best-fitted model explained 75% of the variance (*P*<0.001). The curves for well-watered conditions followed the equations: P_n_ = 17.27 − 0.27 × 1.096^day^ for Bahatans-87 and P_n_ = 13.98 + 11.2 × 0.857^day^ for Tincurrin, indicating that Bahatans-87 had a 22% higher photosynthetic rate than Tincurrin. The curves for terminal drought followed the equations: P_n_ = 2.321 + 100.9 × 0.7465^day^ for Bahatans-87 and P_n_ = 19.71 − 1.4 × 1.1023^day^ for Tincurrin, indicating that Bahatans-87 growth rate declined 32% faster than Tincurrin.

The transpiration rate (T_r_) under well-watered conditions ranged from 3.5 to 5.5 mmol m^−2^ s^−1^ in both cultivars (**Figure 2C**). However, under terminal drought, the transpiration rate declined in Bahatans-87 from 3.4 to 0.3 mmol m^−2^ s^−1^ and Tincurrin from 5.1 to 1.1 mmol m^−2^ s^−1^ in Tincurrin (**Figure 2C**). The best-fitted model explained 81% of the variance (*P*<0.001). Under well-watered conditions, the T_r_ of Tincurrin increased 14% faster than Bahatans-87, while under terminal drought, the Tr of Bahatans-87 declined 41% faster than Tincurrin.

### Plant Growth

**Figure 3:**
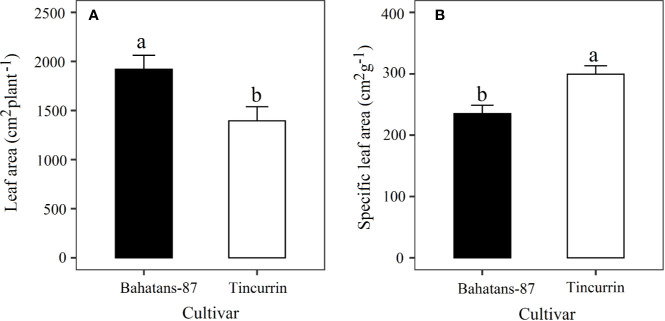
**(A)** Leaf area and **(B)** specific leaf area after the first spikelet on the main stem ear was visible (Z51) in Bahatans-87 (large root system) and Tincurrin (small root system), just before terminal drought was induced. Means followed by different letters differ significantly (*P* < 0.05). Vertical error bars represent s.e.m (n= 5).

**Figure 4:**
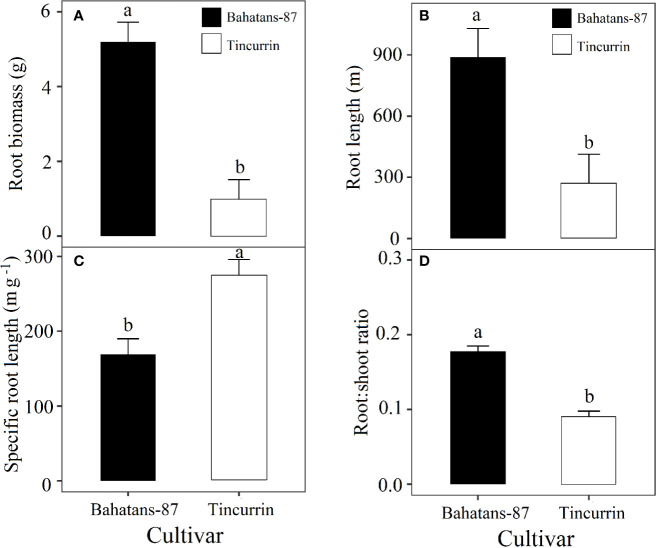
**(A)** Root biomass, **(B)** root length, **(C)** specific root length, and **(D)** root/shoot ratio in Bahatans-87 (large root system) Tincurrin (small root system) after the first spikelet on the main stem ear was visible (Z51), just before terminal drought was induced. Means followed by different letters differ significantly (*P*< 0.05). Vertical error bars represent s.e.m (n= 5).

At the first spikelet on the main stem ear appeared, just before terminal drought was induced, Bahatans-87 had 28% more leaf area than Tincurrin (*P*<0.05; **Figure 3A**) and lower specific leaf area (235 cm^2^ g^−1^) than Tincurrin (299 cm^2^ g^−1^) (*P*<0.01; **Figure 3B**). The specific leaf area provides the leaf thickness values. The higher specific leaf area of Tincurrin indicates its thinner leaves than Bahatans-87. Thinner leaves of Tincurrin can be an advantageous in terms of higher photosynthetic efficiency per unit of leaf area; however Bahatans-87 compensated with a larger leaf area. Bahatans-87 also had 81%, 70%, and 47% higher root biomass, root length, and root: shoot ratio than Tincurrin (*P*<0.05; **Figure 4**), but Tincurrin had 40% higher specific root length than Bahatans-87 (*P*<0.01; **Figure 4C**), indicating that Tincurrin had a thinner root system than Bahatans-87.

**Figure 5:**
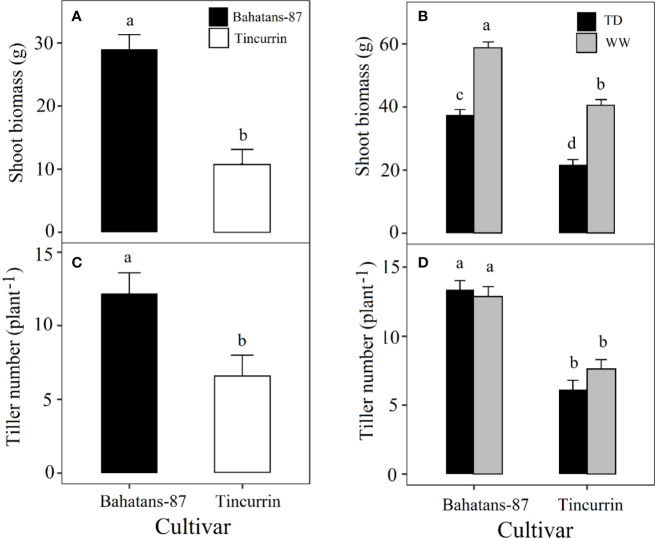
Shoot biomass **(A)**, before the watering treatments were applied and **(B)** at final harvest, and tiller number **(C)** just before terminal drought was induced and **(D)** at final harvest in Bahatans-87 (large root system) and Tincurrin (small root system) under well-watered (WW) and terminal drought conditions (TD) from the time the first spikelet on the main stem ear was visible. Means followed by different letters differ significantly (*P*< 0.05). Vertical error bars represent s.e.m (n= 7).

Shoot biomass, before terminal drought was induced, was 63% higher in Bahatans-87 than Tincurrin (*P*<0.001; **Figure 5A**). At final harvest, Bahatans-87 had 31% more shoot biomass under well-watered conditions than Tincurrin (*P*<0.001; **Figure 5B**) and 42% more shoot biomass under terminal drought conditions than Tincurrin (*P*<0.001; **Figure 5B**). Terminal drought reduced shoot biomass more in Tincurrin (47%) than in Bahatans-87 (36%) (*P*<0.001; **Figure 5B**). Tiller number, before terminal drought was induced, was higher in Bahatans-87 (five more tillers) than Tincurrin (*P*<0.01; **Figure 5C**). At final harvest, Bahatans-87 had seven more tillers than Tincurrin, irrespective of water treatment (*P*<0.001; **Figure 5D**). Terminal drought had no effect on tiller number in either cultivar (*P*>0.05), mainly because it was induced at ear emergence.

### Water Use and Water Use Efficiency

Under well-watered conditions, Bahatans-87 had 45% and 33% greater pre-and post-ear emergence water use than Tincurrin, respectively (*P*<0.01; **Table 2**). Across the whole experiment, under well-watered conditions, Bahatans-87 used 39% more water than Tincurrin. Under terminal drought, post-ear emergence water use decreased by 82% in Bahatans-87 and 49% in Tincurrin, compared with their respective well-watered treatment (*P*<0.01; **Table 2**). Across the whole experiment, under terminal drought, Bahatans-87 used 39% more water than Tincurrin (*P*<0.01; **Table 2**). Terminal drought reduced total water use by 37% in both cultivars (*P*<0.01). The ratio of pre-and post-ear emergence water use was comparable in both cultivars under well-watered conditions, but it almost doubled under terminal drought (*P*<0.001). Tincurrin had 36% and 59% higher WUE_grain_ than Bahatans-87 under well-watered and terminal drought conditions, respectively (*P*<0.001; **Table 2**). Under terminal drought, WUE_grain_ declined by 67% in Bahatans-87 and 49% in Tincurrin (*P*<0.001; **Table 2**).

**Table 2:** Pre- and post-ear emergence water use, total water use, ratio of pre-ear to post-ear emergence water use, and water use efficiency (WUE _grain_), in Bahatans-87 (large root system) and Tincurrin (small root system) under well-watered (WW) and terminal drought conditions (TD) from the time the first spikelet on the main stem ear was visible.

Cultivar	Treatment	Pre-ear emergence water use (L plant^−1^)	Post-ear emergence water use (L plant^−1^)	Total water use (L plant^−1^)	Pre-ear/post-ear emergence water use	WUE _grain_(g grain L^−1^)
Bahatans-87	TD	12.0a	1.8c	13.8b	6.6a	0.29d
	WW	12.1a	10.2a	22.2a	1.3c	0.88b
Tincurrin	TD	6.6b	1.8c	8.5c	3.5b	0.71c
	WW	6.6b	6.8b	13.5b	1.0d	1.38a
*P*-value (LSD)						
Cultivar		***(0.13)	**(1.0)	***(1.1)	***(0.12)	***(0.097)
Treatment		NS	***(1.0)	***(1.1)	***(0.12)	***(0.097)
Cultivar × treatment		NS	**(1.4)	**(1.5)	***(0.16)	NS

Means followed by different letters differ significantly (LSD test). **, *** significant at P < 0.01 and P<0.001, respectively. NS, not significant P > 0.05.

### Grain Yield

Under well-watered conditions, Bahatans-87 and Tincurrin had similar grain numbers and grain yields, despite Bahatans-87 producing five more spikes than Tincurrin (*P*<0.05; **Table 3**). Tincurrin had 30% more grains per spike than Bahatans-87 (*P*<0.001; **Table 3**). Under terminal drought, Tincurrin produced 36% higher grain yield than Bahatans-87 (*P*<0.001). Terminal drought reduced grain yield by 67% in Tincurrin and 80% in Bahatans-87 (*P*<0.001). Under terminal drought, Tincurrin had 33% more grains per spike than Bahatans-87 (*P*<0.001). Terminal drought reduced grain number by 30% in Tincurrin and 57% in Bahatans-87 (*P*<0.001). Under terminal drought, both species had similar spike numbers, despite terminal drought reducing spike number per plant by five in Bahatans-87 and one in Tincurrin (*P*<0.05). Tincurrin produced 30% more grains per spike than Bahatans-87 under well-watered conditions (*P*<0.001). Under terminal drought, Bahatans-87 produced 43% more grains per spike than Tincurrin (*P*<0.001). Terminal drought reduced grain number per spike by 31% in Bahatans-87 and 15% in Tincurrin (*P*<0.001). Both cultivars had similar 1000-grain weights (~ 41 g) under well-watered conditions. Terminal drought reduced 1000-grain weight in both cultivars by about 53% (~19 g; *P*<0.001). Under well-watered conditions, Tincurrin had 28% higher HI than Bahatans-87 (*P*<0.001; **Table 3**), which increased to 61% higher under terminal drought (*P*<0.001). Terminal drought reduced HI by 67% in Bahatans-87 and 39% in Tincurrin (*P*<0.001).

**Table 3:** Grain yield, grain number, spike number, grains per spike, 1000-grain weight, and harvest index (HI) in Bahatans-87 (large root system) and Tincurrin (small root system) under well-watered (WW) and terminal drought conditions (TD) from the time the first spikelet on the main stem ear was visible.

Cultivar	Treatment	Grain yield (g plant^−1^)	Grains plant^−1^	Spikes plant^−1^	Grains spike^−1^	1000 grain weight (g)	Harvestindex
Bahatans-87	TD	3.9c	209c	7.9b	26.5d	19.5b	0.11d
	WW	19.4a	484a	12.8a	38.2c	40.3a	0.33b
Tincurrin	TD	6.1b	312b	6.9b	46.2b	19.3b	0.28c
	WW	18.6a	443a	8.1b	54.5a	42.0a	0.46a
*P* value (LSD)							
Cultivar		NS	NS	***(1.4)	***(4.6)	NS	***(0.03)
Treatment		***(1.6)	***(48)	***(1.4)	***(4.6)	***(2.4)	***(0.03)
Cultivar × treatment		NS	**(67)	*(1.9)	NS	NS	NS

Means followed by different letters differ significantly (LSD test). *, **, *** significant at P < 0.05, P < 0.01 and P < 0.001, respectively. NS, not significant P > 0.05.

## Discussion

### Cultivar With Large Root System Size Depleted Soil Water Faster Than Cultivar With Small Root System Size

Before the watering treatments were imposed at ear emergence, the cultivar with the larger root system size (Bahatans-87) had higher leaf area and shoot biomass than the cultivar with smaller root system size (Tincurrin), confirming that the size of the root system in wheat is positively correlated with leaf area and shoot biomass (Liao et al., 2006; Aziz et al., 2016). In near isogenic lines of durum wheat with large root system had great leaf area and biomass at anthesis than the small root system line (Pooniya et al., 2019). Since leaf area and shoot biomass are positively associated with transpiration (Araya et al., 2019), it was expected that Bahatans-87 would deplete available soil water faster than Tincurrin. Indeed, withholding watering from when the first spikelet was visible rapidly reduced soil water content in Bahatans-87 (large root system) 17 days earlier than Tincurrin (small root system), which reflected the faster reduction in stomatal conductance and leaf net photosynthesis. Stomatal conductance dropped below 200 mm m^−2^ s^−1^ within 10 days of withholding water in Bahatans-87, while Tincurrin reached a similar value within 17 days of withholding water, indicating that the cultivar with the large root system size closed the stomata and developed plant water deficit earlier than cultivar with small root system.

### Water Use Higher in the Cultivar With Larger Root System Size

The higher pre-ear emergence water use in Bahatans-87 could be related to a higher demand for water due to its higher leaf area and shoot biomass and slower phenological development. Bahatans-87 (large root system) reached anthesis 23 days after Tincurrin (small root system), confirming that there is a correlation between time to anthesis and root system size in wheat (Aziz et al., 2016; Figueroa-Bustos et al., 2018). In wheat roots reach maximum biomass by anthesis (Gregory et al., 1978). In our experiment, terminal drought stress was imposed at heading when the root system size was fully developed. We did not observe differences in the root system size within a cultivar under the water treatment (well-watered and terminal drought treatment). It is likely that wheat cultivars with longer time to anthesis have more time for root system growth, since the allocation of daily photosynthates to roots decreased abruptly from 42% to 18% at floral initiation (double ridge, Z31) and to 4% by booting (Z47) (Gregory and Atwell, 1991; Palta and Gregory, 1997). The 33% higher post-ear emergence water use in Bahatans-87 than Tincurrin are directly associated with its shoot biomass and higher leaf area, since leaf area and water transpired are linearly correlated (Ritchie, 1974).

### Cultivar With Larger Root System Had Higher Reduction in Grain Yield Than Cultivar With Smaller Root System Under Terminal Drought

The wheat cultivars with contrasting root system size in this study had no significant difference in grain yield under well-watered conditions. Despite both cultivars having similar grain numbers per plant and thousand grain weights, Bahatans-87 had more spikes per plant than Tincurrin. Tincurrin compensated for the lower number of spikes by producing more grains per spike. Bahatans-87 is an old cultivar released in 1924, while Tincurrin was released 54 years later (Figueroa-Bustos et al., 2018). Old cultivars produced more tillers than newer cultivars (Siddique et al., 1989; Fang et al., 2017). However, in dryland environments, many of these tillers die before producing a spike (Duggan et al., 2000). Modern cultivars have more grains per spike than older cultivars (Álvaro et al., 2008; Fang et al., 2017; Qin et al., 2018). Hence, modern cultivars like Tincurrin produce similar grain yields to old cultivars like Bahatans-87 by increasing grain numbers per spike under well-watered conditions.

Terminal drought reduced grain yield in both cultivars, with the differences reflected in phenological development. Bahatans-87 had slower phenological development allowing root and shoot growth to continue for longer (Figueroa-Bustos et al., 2019). Early flowering allows grain filling to be completed before the onset of severe water stress (Shavrukov et al., 2017). In Mediterranean environments cultivars with slower phenological development had higher pre-anthesis water use and less soil water available for reproductive stages (Siddique et al., 1990b). Post-anthesis water use is directly used for grain filling, such that cultivars that used more water during vegetative stages end up with grain yield penalties (Fischer and Wood, 1979). Hence, the faster phenology, lower ratio of pre-to post-ear emergence water use, and lower total water use in Tincurrin under terminal drought might explain its smaller reduction in grain yield, relative to Bahatans-87.

### Grain Number Affected Yield in Cultivar With Larger Root System Size Under Terminal Drought

The critical period for grain set in wheat is from the appearance of the penultimate leaf (Z33) to the beginning of grain filling 10 days after anthesis (Z65) (Zadoks et al., 1974; Fischer, 2008). Soil water content at 10 days after flowering, when grain number was already set, was 27% in Bahatans-87 and 34% in Tincurrin, indicating that Bahatans-87 depleted most of the available soil water before grain number was determined. Since terminal drought reduced photosynthesis in Bahatans-87 earlier and faster than Tincurrin, grain number declined significantly in Bahatans-87, presumably as current photosynthate is essential for maintaining grain number during the critical period for grain set in wheat (Kirby, 1988). It is also likely that reduction in photosynthesis induces floret abortion, reduction in grain number, and grain filling (Rajala et al., 2009).

### Cultivar With Smaller Root System Size Had Better Grain Water Use Efficiency

The higher water use efficiency of the cultivar with smaller root system size (Tincurrin) was associated with its low ratio of pre- to post-ear emergence water use, longer grain filling duration, and higher harvest index than the cultivar with the larger root system size (Bahatans-87). Selection for high yield in wheat has also lowered the ratio of pre- to post-anthesis water use, indicating that modern wheat cultivars use proportionally less water during vegetative stages, conserving water in the soil for reproductive stages (Siddique et al., 1990b). The lower ratio of pre- to post-anthesis water use and lower total water use in Tincurrin is likely due to its early anthesis and lower shoot biomass than Bahatans-87. Tincurrin is a semi-dwarf cultivar with lower stem weight that could increase harvest index (Siddique et al., 1989; Acreche et al., 2008; Friedli et al., 2019). Increases in harvest index are associated with increases in grain yield (Perry and D’Antuono, 1989; Richards et al., 2014).

Breeding programs for improving wheat grain yield in dry environments and dry seasons have mainly focused on improving tolerance to terminal drought (Siddique et al., 1990a; Asseng and van Herwaarden, 2003; Golabadi et al., 2010; Senapati et al., 2018) by selecting for early vigor and early flowering to minimize frost damage and escape the effects of terminal drought (Perry and D’Antuono, 1989; Rebetzke and Richards, 1999; Richards et al., 2014). This advantage seems to diminish when cultivars selected for early anthesis experience early season drought, which main effect is to delays phenology, particularly time to anthesis (Figueroa-Bustos et al., 2019). With the slow onset of climate change, early winter rainfall in the Mediterranean-type climate of Australia has been decreasing; as a result, more than 82% of wheat growers are dry sowing their crops (Fletcher et al., 2015). Wheat crops sown into dry soil will germinate on the first rainfall, potentially leaving crops vulnerable to 20-32 days of drought after emergence, called early season drought (French and Palta, 2014).

Despite the fact that several studies indicated that increasing root length and root biomass could associate with drought tolerance on field conditions (Heřmanská et al., 2015; Palta and Turner, 2018); the five decades of breeding and selection for yield showed a reduction in root length and root biomass in modern wheat compared to older cultivars (Siddique et al., 1990a; Aziz et al., 2016; Fang et al., 2017). This is due to the strong positive association between root system size, leaf biomass, and phenology that increases water use which in turn reduces water use efficiency and yield in plants under terminal drought.

This study was conducted in a controlled environment with two wheat cultivars differ in root system size. To validate our findings further studies with more cultivars are required under field conditions. Differences in root system size measured in the glasshouse also needs validation under field conditions; since the growth of the root system in wheat depends on a number of factors and their interaction, such as such as soil type (clay vs. sand), soil physical and chemical characteristics, and the soil water content that, in turn, is influenced by the amount and distribution of rainfall. Root models such as ROOTMAP (Diggle, 1988) and OpenSimRoot (Postma et al., 2017) provide useful tool to not only to validate root traits and root system architecture, but also allow to compute root acquisition of water and nutrients (Chen et al., 2011; Chen et al., 2013). The modeling simulation has the potential to elucidate the role of the root system in conferring tolerance to terminal drought (Dunbabin et al., 2013).

## Conclusion

Differences in grain yield between wheat cultivars with contrasting root system size under terminal drought, were mainly related to water use, particularly post-ear emergence water use. Bahatans-87 (larger root system) depleted the available soil water faster than Tincurrin (smaller root system) due to higher leaf area and shoot biomass. Under well-watered conditions, both cultivars had similar grain yields, despite, Tincurrin having higher water use efficiency. Under terminal drought, leaf net photosynthesis rate during the first 10 days after anthesis sharply declined in Bahatans-87, which significantly affected grain number per plant, while the slow reduction in soil water content and photosynthesis in Tincurrin resulted in smaller reductions in grain number per plant. Terminal drought reduced grain yield in both cultivars, more so in Bahatans-87 than Tincurrin. The strong association between root system size and phenology, leaf area, and shoot biomass, determined cultivar performance under terminal drought. Further studies to improve grain yield in water-limited environments should consider that association.

## Data Availability Statement

The original contributions presented in the study are included in the article or supplementary material; further inquiries can be directed to the corresponding author.

## Author Contributions

VF-B, JP, YC, and KHMS conceived and designed the experiments. VF-B performed the experiments along the supervisions of JAP, YC, and KHMS. VF-B and KS analyzed the data. VF-B wrote the manuscript. JP, YC, and KHMS revised the manuscript.

## Funding

VF-B acknowledges The National Commission for Scientific and Technological Research of Chile (CONICYT) and The University of Western Australia’s Institute of Agriculture and School of Agriculture and Environment for funding this research.

## Conflict of Interest

The authors declare that the research was conducted in the absence of any commercial or financial relationships that could be construed as a potential conflict of interest.

## Supplementary Material

Figure S1 Relationship between fraction of transpirable soil water and the ratio of water stressed to well-watered for **(A)** stomatal conductance, **(B)** photosynthesis rate, and **(C)** transpiration rate in two wheat cultivars Bahatans-87 and Tincurrin.
