# Supplementary material for: Wheat Cultivars With Contrasting Root System Size Responded Differently to Terminal Drought
Source: Front Plant Sci. 2020 Aug 19;11:1285. doi: 10.3389/fpls.2020.01285 (PMC7466772; doi:10.3389/fpls.2020.01285)
Supplement: Figure S1 — Relationship between fraction of transpirable soil water and the ratio of water stressed to well-watered for (A) stomatal conductance, (B) photosynthesis rate, and (C) transpiration rate in two wheat cultivars Bahatans-87 and Tincurrin. [file Image_1.pdf]

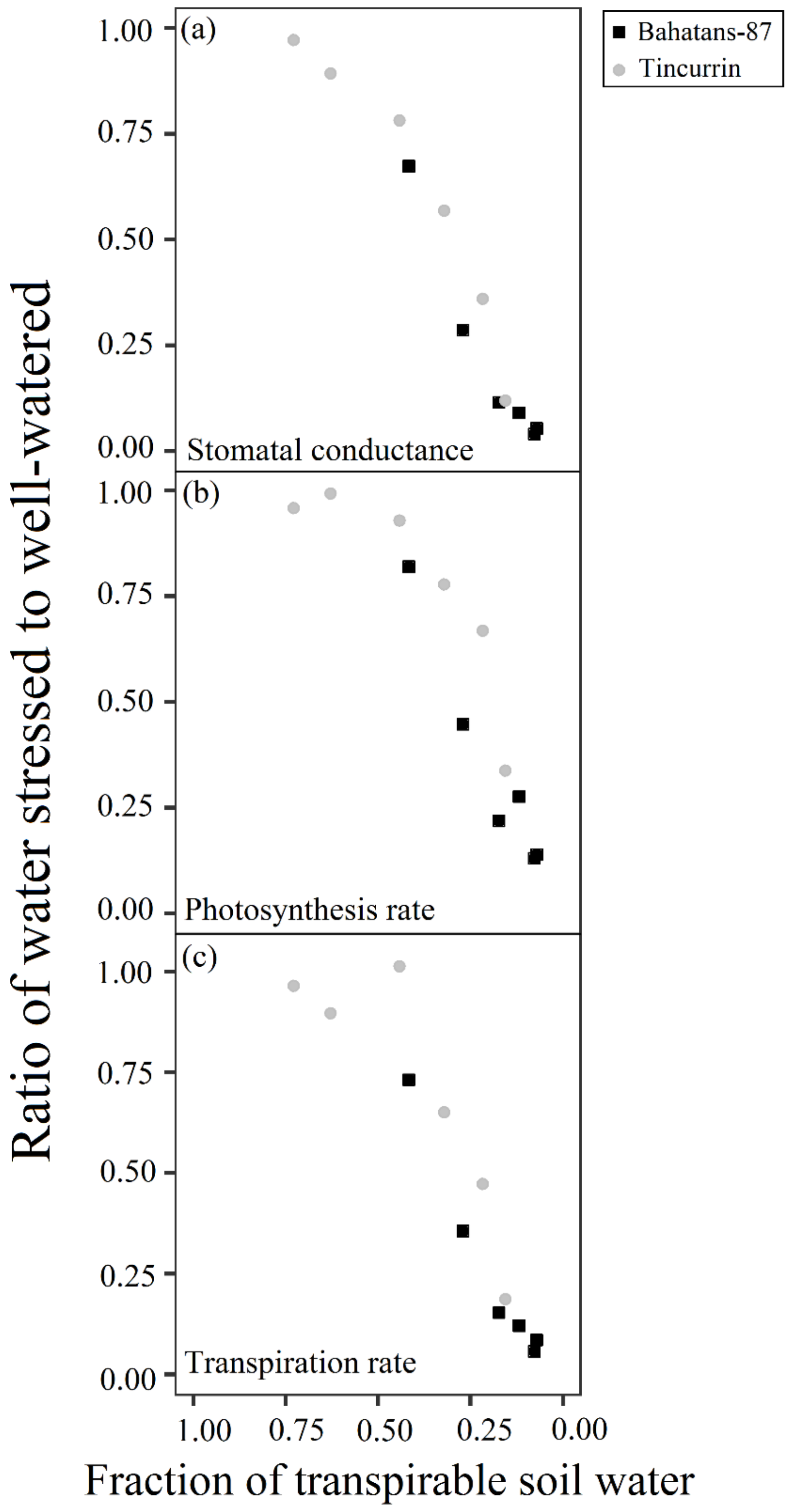

Figure 1S. Relationship between fraction of transpirable soil water and the ratio of water stressed to well-watered for (a) stomatal conductance, (b) photosynthesis rate and (c) transpiration rate in two wheat cultivars Bahatans-87 and Tincurrin.
